# Not all engaged students are alike: patterns of engagement and burnout among elementary students using a person-centered approach

**DOI:** 10.1186/s40359-023-01071-z

**Published:** 2023-02-09

**Authors:** Dong Yang, Zhenyu Cai, Chaoyi Wang, Chen Zhang, Peng Chen, Ronghuai Huang

**Affiliations:** 1 Smart Learning Institute, Beijing Normal University, Beijing, China; 2 College of Child Development and Education, Zhejiang Normal University, Hangzhou, China; 3 College of Education, Capital Normal University, Beijing, China

**Keywords:** Latent profile analysis, Student engagement, School burnout, Motivational styles, Teacher behaviors

## Abstract

Due to its potential to address low achievement, high dropout rates, and misbehavior among students, school engagement has become an important topic in contemporary developmental psychology and educational research. Although there is a wealth of literature on the causes and effects of student engagement, the current understanding of how student engagement varies in response to different teaching styles is limited. This study examined the engagement and burnout profiles of elementary school pupils (N = 798; 51% females; M_age_ = 11.54, SD_age_ = 0.72) and the interactions between those profiles, students’ characteristics and their perceptions of instructional behaviors (e.g., supporting criticism, suppressing criticism & independent viewpoints, intruding). Latent profile analysis revealed five types of profiles: moderately burned out, slightly burned out, moderately engaged, highly engaged, and highly burned out. Follow-up logistic regression analysis found that students clustered into engagement groups were likely to report higher autonomy support from teachers, especially when teachers permit criticism and independent thinking from students. In contrast, students clustered into burned out profiles were more likely to rate teacher strategies as autonomy suppressive. This became more obvious when instructors imposed meaningless and uninteresting activities. Taken together, this study indicated that autonomy-supportive teaching behaviors are pivotal in understanding student engagement and school burnout. The significance of the findings was addressed, along with implications and limitations.

## Introduction

Student engagement is strongly linked with numerous desired academic and nonacademic outcomes, such as learning achievement and physical and psychological well-being [1, 2]. Increasing students’ engagement in learning is also an essential objective for educators. As a result, practitioners, policy-makers, and scholars continue to use engagement as a key target for interventions and as an explicit goal of many school improvement programs [e.g., 2–4]. Student engagement has been characterized as a student’s commitment and investment in schooling [5]; time and energy students devote to educational activities [6]; or even positive emotions and learning strategies [7]. Despite variations in how engagement is defined, there seems to be a consensus that it is a multidimensional construct that integrates behavior, cognition, and emotion [5]. One study [8] applied the idea of work engagement to the school context, defining schoolwork engagement from the viewpoints of energy, dedication, and absorption. Within this framework, energy and dedication refer to high energy and resilience while undertaking school-related responsibilities, whereas absorption depicts immersion in studies or hobbies [9].

On the other hand, because of their importance in predicting low academic success, student misbehavior, and school dropout, recent developmental psychology and educational psychology studies have started to investigate school burnout in students alongside engagement [1, 10]. They realized that it is essential to simultaneously examine and simulate both the benefits and drawbacks of engagement [11]. As defined by scholars [12], school burnout occurs when students feel emotionally exhausted (e.g., overwhelmed by schoolwork), cynical and detached from their academic interests (e.g., experiencing a loss of meaning in studying), and inadequate (e.g., believing their capacities to be lacking). Exhaustion of emotion is a symptom of a psychiatric condition similar to the emotional state of disengagement. Burnout symptoms in academic settings can be produced by high perceived study demands, a cynical and detached attitude toward one’s studies, or feelings of inadequacy [12–14].

A variety of age groups and settings have made use of the concepts of school engagement and burnout [15]. However, the majority of studies exploring this topic were undertaken in secondary settings [e.g., 16–19, 20], with limited research conducted at the elementary school level. For example, a previous study investigated Finnish high school students’ latent profiles of school burnout symptoms, studyholism, and engagement and how socioemotional skills predicted those profiles [21]. Another study examined the relationship of degrees of burnout and school engagement with students’ academic success, study habits, and self-efficacy beliefs among Turkish secondary school students [22]. However, empirical evidence has shown that students’ susceptibility to burnout symptoms is increased after transitioning to secondary school [23]. From a developmental perspective, early screening of such symptoms is crucial even before the secondary school stage. Accumulating evidence suggests that engagement is an important factor to consider in elementary school, as it is a robust predictor of long-term academic achievement outcomes [3]. Moreover, the majority of these studies were conducted in Europe, especially in Scandinavia [9, 24–26], and there is currently a lack of evidence of school burnout in China (a typical Eastern culture).

Previous research has investigated the predictive role of social support (e.g., parents, teachers, and peers) on profiles of student engagement [7, 19] from a macro perspective. However, no studies have explored how different forms of supportive teaching behaviors are linked with engagement and burnout profiles. In this study, we moved one step further and tested how various students’ perceived teaching behaviors contribute to their engagement and burnout profiles.

Unlike variable-centered analyses, person-centered research begins with the idea that individual differences may reflect subpopulations and that a model based on the average population cannot apply to all individuals [27]. The person-centered approach could be used to overcome heterogeneity [28] and to determine why some pupils adapt well to school while others do not. For instance, a student can be highly engaged but experience a high degree of school burnout [8]. Using the intriguing findings from this area of study as inspiration and built on recent work [17, 19], we used a person-centered approach among a sample of elementary school students to identify profiles resulting from the interaction of engagement and burnout; we then investigated students’ perceptions of autonomy support by examining associations between the profiles aspects of autonomy support (controlling vs. autonomy supportive) and student characteristics (i.e., gender, school regions, grade).

This study contributes to the current literature by examining the engagement and burnout profiles in urban and suburban contexts. Moreover, this work is different from previous work that focused heavily on middle and high school students [29]. Using a person-centered approach, we identified the patterns of student engagement/burnout using responses from an elementary student population (grades 4 to 6). From the perspective of developmental psychology, both school burnout and student engagement are indicators of a student’s social and emotional health and may shed light on their psychological well-being and academic adjustment [19].

## Literature review

### Engagement, burnout among students, and the role of autonomy support

In a broader sense, school engagement and burnout can be defined as the quantity and quality of students’ involvement and disengagement in school or school activities [27]. Furthermore, engagement and burnout are multifaceted and feature dynamic and bilateral processes sensitive to the learning environment [5, 30]. Engaged students are more likely to earn higher grades and better personal adjustment to school. Additionally, burned out students are more likely to fail academically, drop out of school, and be exposed to various negative psychosocial consequences [31]. Moreover, scholars have agreed that school engagement and burnout are malleable states that can be shaped by school context [4, 32]. Some studies have revealed, however, that the experience of energy, dedication, and absorption inherent in student engagement often coincides with negative emotions such as tiredness or inadequacy, which are common indications of student burnout [25].

The importance of student burnout in the classroom has been recognized in recent years [33], as symptoms may worsen with employment. Initially, burnout was defined as “feeling exhausted because of study demands, having a cynical and detached attitude toward one’s study, and feeling incompetent as a student” [13]. Quite recently, based on the rationale that “school is a place where students work” [34], the three-component construct of burnout was extended to the school context ([9]; see [35] for a review). In this sense, burnout might be seen as an inability to meet performance expectations. Signs of this condition include exhaustion, a cynical attitude toward schoolwork, and a lack of confidence [30, 36]. When previous studies [9, 11] examined the relationship between engagement and burnout in the context of studies with high school students, a wide range of student profiles displaying varying degrees of both variables emerged. Some students reported low levels of burnout and high levels of engagement in their schoolwork, whereas others reported high levels of burnout and low levels of engagement. Such findings run counter to the conventional understanding of engagement as an unquestionably good experience and have significant clinical consequences because engaged-exhausted students are at increased risk for developing depressive symptoms over time [15, 20].

Burnout in the classroom might be viewed as a symptom of disengagement in one’s studies [15]. Despite their negative correlation, school disengagement and burnout are distinct concepts. Generally, students’ disinterest in their studies increases proportionally to the number of burnout symptoms they report. Nevertheless, the absence of such symptoms does not necessarily indicate an increase in interest. According to one study [15], some high school pupils displayed both enthusiasm and exhaustion. Other studies [31] found that there were distinct long-term relationships between students’ levels of engagement and burnout at school and their academic and mental health. As a result, unique psychological mechanisms are at play in the growth of school-related outcomes related to the negative emotional processes associated with burnout. In addition, it seems to be a trend to study students’ profiles of engagement and burnout in relation to different psychological/motivational factors. For example, degrees of burnout and school engagement in relation to academic success, study habits, and self-efficacy beliefs [22] found that levels of burnout were higher in pupils with low self-efficacy. Students with adequate study skills and high self-efficacy beliefs were likewise highly engaged in school. Some studies went further and investigated high school students’ latent profiles of school burnout symptoms, engagement, and studyholism and how socioemotional skills predict students’ membership in different latent groups. This study demonstrated for the first time that socioemotional resources can mitigate school burnout [21]. Moreover, scholars are also interested in how student engagement and burnout symptoms are linked to academic achievement [15, 37].

Little is known about how students’ engagement (and/or burnout) is connected to their impressions of the school’s atmosphere. Nonetheless, the results of the few extant studies in the field are intriguing. Prior research has shown that it negatively correlates with students’ levels of support [38, 39]. Some researchers have investigated the role of supportive sociocontextual factors in building students’ academic engagement [40]. One such aspect of support is students’ perceived teaching practice, which was generally conceptualized as a stable pattern in a teacher’s methods of instruction, classroom management, and interpersonal style with students [41].

Regarding teaching styles, a teacher can be either supportive or suppressive [42]. Over the last two decades, ample evidence suggests how different teaching practices (e.g., motivational styles) could predict student engagement and burnout at school [43, 44]. For example, both positive (e.g., providing choice, fostering understanding and interest) and negative indicators (e.g., intruding, suppressing criticism & independent opinions) of teacher motivational styles were studied across different subjects [43–46]. A cross-cultural study that compared American and Chinese pupils found that a significant relationship between school atmosphere and engagement existed in only one American sample [47]. These findings suggest that further investigation into the correlation between classroom atmosphere (e.g., teaching styles) and student engagement/burnout is warranted. However, research into the links between the school environment and student burnout has been quite limited. One study [48] showed a negative correlation between students’ perceptions of classroom structure and burnout among the few studies conducted in this area. Knowing the extent to which young pupils feel and act in school is related to school climate can inform interventions for enhancing the learning environment. Hence, research in this field has substantial educational implications. More recently, a person-centered study that employs latent profile analysis (LPA) found that middle school students with different profiles also varied in their perceptions of the school’s climate [20].

### The interactions between student characteristics and engagement profiles

Although student burnout and engagement are mostly negatively correlated, this does not mean that they cannot coexist in various combinations. Earlier person-oriented studies [9, 11, 15] described profiles of students indicating high levels of engagement alongside exhaustion and cynicism. Burnout among students is a serious problem, as studies have shown that it can lead to depression, anxiety [12], and school withdrawal [49]. While most research on such topics is limited, it is clear that understanding the factors that contribute to a positive school climate and prevent students from becoming burned out is crucial for informing effective strategies to foster well-being and productive classroom environments.

Multiple student demographic characteristics, such as gender and grade, and other contextual factors, such as urban schooling, are meaningfully related to students’ engagement and burnout in school [35]. Regarding gender, various studies have demonstrated gender differences across contexts. For example, gender was associated with school burnout symptoms, independent of grades; girls reported a significantly higher level of exhaustion in the study of [24], and girls’ greater academic performance, higher grades, greater frequency of enrolling in advanced courses, and supposedly higher levels of discipline and engagement may come at the expense of exhaustion and waning motivation [4, 15]. In addition to gender, grade level is also an important student-level factor, as declines in motivation and engagement begin to emerge in late elementary school and continue into secondary educational experiences [50–52]. Finally, disengagement and disconnection from school experiences may be heightened for students attending schools in urban settings due to a confluence of contextual factors such as larger student–teacher ratios, inequitable learning opportunities, misalignment between the academic curriculum and students’ cultural identities and values, and chronic risk factors (e.g., health disparities, lack of resources) associated with poverty [53, 54]. Collectively, the literature shows the importance of individual and contextual factors on students’ engagement and burnout during the primary school years.

## Methods

### Sample and procedure

This study involved 798 participants (51.5% females; two students did not reveal their gender) from four mixed-gender elementary schools in a large southwest Chinese province. Participants were 9 to 13 years (M_age_ = 11.54, SD_age_ = 0.72) and were all in the 4th 5th, or 6th grades. A total of 48.4% (N = 386) of students were from suburban schools, and 51.6% (N = 412) of students were identified as attending urban school based on the classification of the Chinese National Bureau of Statistics (CNBS; www.stats.gov.cn). Their socioeconomic status (SES) was not assessed directly for this study, but the official website of the CNBS reveals that participating schools are attended by students from both low and medium socioeconomic contexts. The questionnaire included students’ demographic information (i.e., age, gender, grade), self-reported school engagement, burnout, and perceived teacher autonomy support. Before participating in the survey, an informed consent form was sent to students and guardians. Participation was voluntary, and only students whose parents gave their written informed consent could participate in the study. Participants were fully aware of the purpose of the study and data management plan before they agreed to continue. The study was conducted in June 2022. Data were collected using a pencil-and-paper form questionnaire with help from class teachers. On average, students were given 15 min to finish the questionnaire after a certain class. The study protocol follows the Declaration of Helsinki of 1964 and its latest versions and was approved by the first author’s University Ethical Review Board in the Humanities and Social and Behavioral Sciences.

### Measures

#### Student engagement

The Schoolwork Engagement Inventory (SEI; [8]) was used to measure student engagement. The SEI is a validated scale used in many previous studies [8, 55, 56]. Specifically, the SEI consists of nine items that measure student engagement via vigor/energy (three items. e.g., when studying, I feel strong and vigorous), dedication (three items. e.g., I am enthusiastic about my studies/work), and the state of absorption (three items. e.g., I feel happy when I am studying intensively). The replies were scored on a four-point scale (1 = very untrue of me; 4 = very true of me), and a total score (Cronbach’s = 0.93) was computed to measure the student’s overall school participation.

#### Burnout

Burnout was measured by the School Burnout Inventory (SBI; [25]). Responses were rated on a four-point scale (1 = very untrue of me; 4 = very true of me). Like the SEI, the SBI is a validated, three-component scale for measuring burnout in a school context (e.g., [15], see [25] for reliability and validity). The scale consists of 10 items measuring three aspects of school burnout: exhaustion at school (e.g., “I feel overwhelmed by my schoolwork”), cynicism toward the meaning of school (e.g., “I feel that I am losing interest in my schoolwork”), and a sense of inadequacy at school (e.g., “I often have feelings of inadequacy in my schoolwork”), all of which are indicative of school burnout. In this study, we were interested in identifying student engagement and burnout profiles based on dimensions of burnout. Thus, Cronbach’s alphas for each dimension were calculated separately (.91 for exhaustion, .88 for cynicism, and .89 for inadequacy).

#### Instructional behaviors

To measure students’ perceived teaching styles, we used validated questionnaires from a prior study [43] to quantify autonomy-enhancing and autonomy-suppressing instructor behaviors. (1) providing choice (e.g., When teacher gives us an assignment, she allows us to choose which questions to answer); (2) cultivating understanding and interest (e.g., teacher explains why it is important to study certain subjects in school); and (3) permitting criticism and encouraging independent thought. (e.g., teacher is willing to listen to students’ complaints regarding her); (4) suppressing criticism and independent viewpoints (e.g., teacher acts in a vindictive way toward students who oppose her opinions).); (5) imposing meaningless and uninteresting activities (e.g., teacher makes me read dull things, i.e., books, stories or instructions); and (6) intruding (e.g., teacher is strict about me doing everything in her way). Each component had three items (the sum of 1, 2, and 3 for autonomy support and the sum of 4, 5, and 6 for autonomy suppression). Students’ replies were scored on a 4-point scale (1 = strongly disagree, 4 = strongly agree), and the Cronbach alpha coefficients or each dimension of teacher behavior were .78, .82, .80, .85, .90, and 0.81, respectively. Table 1 shows the correlations between variables as well as the descriptive statistics.

**Table 1:** Pearson’s correlations among all variables

Variable	1	2	3	4	5	6	7	8	9	10
1. ENG	–									
2. EXH	− 0.42***	–								
3. CYNI	− 0.49***	0.79***	–							
4. INAD	− 0.34***	0.66***	0.65***	–						
5. CHOI	0.51***	0.221***	0.22***	− 0.17***	–					
6. UNDE	0.47***	− 0.27***	0.32***	− 0.24***	0.68***	–				
7. CRIT	0.52***	− 0.28***	0.28***	0.235***	0.66***	0.70***	–			
8. INTR	− 0.02	0.22***	0.19***	0.151***	0.015	0.004	− 0.036	–		
9. SUPR	− 0.24***	0.43***	0.45***	0.30***	0.22***	0.32***	0.36***	0.45***	–	
10. MEAN	− 0.31***	0.51***	0.48***	0.35***	0.25***	0.34***	0.34***	0.33***	.70***	–
M	29.31	7.50	5.37	4.30	9.79	10.39	10.14	7.42	4.91	5.02
SD	5.67	2.83	2.15	1.57	1.95	1.75	1.88	1.88	2.21	2.32

*ENG* Engagement; *EXH* Exhaustion; *CYNI* Cynicism; *INAD* Inadequate; *CHOI* Providing choices; *UNDE* Cultivating understanding and interest; *CRIT* Permitting criticism and independent thought; *INTR* Intruding; *SUPR* Suppressing criticism and independent viewpoints; *MEAN* Imposing meaningless and uninteresting activities

***p < .001

### Analysis plan

All the analyses were performed with the Mplus statistical package (version 8; L. Muthén & Muthén, 1998–2017) with the missing data method, which enabled us to use all the observations in the dataset without inputting the missing data. To identify the homogeneous latent groups of students with different levels of exhaustion, cynicism, feelings of inadequacy, and engagement in studies, we conducted a latent profile analysis with the 3-step approach [57]. We analyzed their associations with teacher autonomy support dimensions and student characteristics (gender, grade, and school region) while controlling for measurement errors in identifying latent classes.

The latent profile analyses were carried out with standardized values of school burnout (e.g., exhaustion, cynicism, and feelings of inadequacy) and engagement in two phases. First, we used student engagement and the three dimensions of school burnout to test models with 2–5 latent classes. Models were then compared using several fit indices: the Bayesian Information Criterion (BIC), the Vuong–Lo–Mendell–Rubin Likelihood Ratio Test (VLMR-LRT), the Bootstrapped Likelihood Ratio Test (BLRT), and the entropy value [58]. The estimation started from a one-class solution to estimate the parameters for 2,3, …, k-class solutions. The solution that best fit the data and seemed reasonable in interpretation was chosen as the final latent profile model. Second, to identify the possible predictors of students’ engagement and burnout profiles, perceived autonomy support and student characteristics variables (gender, grade, and school region) were added to the final LPA model as covariates. Each covariate was added to the model separately. Correlations, means, and variances for all the examined variables are presented in Table 1.

## Results

### Latent profiles of elementary school students based on engagement and burnout

One to six profile solutions were evaluated to determine the most effective model. Adjustment indicators (see Table 2) demonstrated that the five-profile approach provided a better match for the data, since entropy was near 1 in this five-profile model, and the AIC, BIC, and adjusted BIC were lower than in earlier models. The likelihood of correctly identifying students in each profile ranged from 0.83 to 0.94. In addition, the five-profile solution was the final model in which the adjusted-VLMR LRT was significant, indicating that this model fits the data better than the three-profile and six-profile solutions. In addition, the six-profile solution yielded no additional profile. Collectively, the five-profile solution is more parsimonious [59].

**Table 2:** Fit indices for the compared mixture models

No. of Profiles	FP	LL	AIC	BIC	SABIC	Entropy	ALMR(p)	BLRT(p)	Smallest Proportion
1	8	− 7713.18	15,442.35	15,479.81	15,454.40	–	–	–	–
2	13	7219.05	14,464.09	14,524.96	14,483.68	0.83	0.0000	0.0000	44.11%
3	18	6922.12	13,880.25	13,964.53	13,907.37	0.91	0.0000	0.0000	7.14%
4	23	6809.84	13,665.68	13,773.37	13,700.33	0.94	0.0000	0.0000	3.89%
5	28	6721.06	13,498.11	13,629.21	13,540.30	0.92	0.0001	0.0000	3.89%
6	33	6681.02	13,428.05	13,582.56	13,477.76	0.94	0.0056	0.0000	2.50%

*FP* Free parameters, *LL* Log likelihood value, *VLMR(p)* *p* value for VLMR test, *BLRT(p)* *p* value for BLRT test

**Fig. 1:**
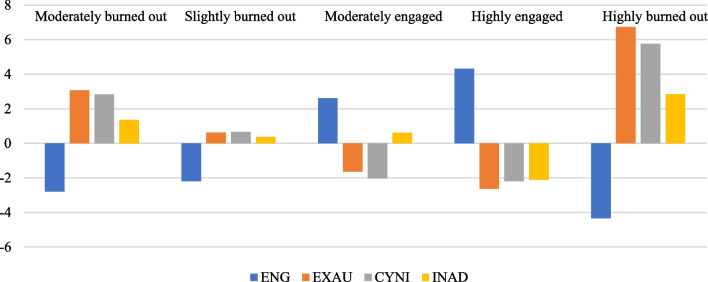
Standardized means on the clustering variables by profiles

As illustrated in Fig. 1, the five-profile solution was generated from the LPA.

The first profile, consisting of 12% (n = 96) of the students, was labeled *moderately burned out*. This group yielded below-average profile means on engagement and a moderately high level of means above the grand means across three indicators (exhaustion, cynicism, and inadequacy) of burnout. The second profile is the largest subgroup (44%, n = 351). It was labeled *slightly burned out*, as it manifested with a below-average level of student engagement and slightly exceeded the grand mean for each indicator burnout on all indicators. A subgroup of 15% (n = 120) of the study was labeled *moderately engaged*. This profile stood out for its relatively high level of engagement and lo, lower-than-average exhaustion and cynicism, along with a higher-than-average level of inadequacy. In addition, the fourth profile, which consisted of a quarter of the population (25%, n = 199), was named *highly engaged* since it showed a fourfold higher than the grand mean level on student engagement, while the lowest level of means was below the average across all three dimensions of burnout symptoms. The last subgroup is also the smallest, containing 4% (n = 32) of the students, featuring an extraordinarily high level of exhaustion (7 times higher), cynicism (nearly 6 times higher), and inadequacy (3 times higher). This group was labeled *highly burned out.* In addition to the profiles, we also presented the descriptive data of unstandardized final latent profile solutions; Table 3 shows more details.

**Table 3:** Unstandardized final latent profile solution

	Engagement		Exhaustion		Cynicism		Inadequacy	
	M	SD	M	SD	M	SD	M	SD
Moderately burned out	26.52	5.28	10.57	1.79	8.20	0.74	5.65	1.11
Slightly burned out	27.12	4.66	8.13	1.64	6.04	0.66	4.66	0.94
Moderately engaged	31.93	4.27	5.86	2.08	3.34	0.54	4.90	0.83
Highly engaged	33.63	4.04	4.87	1.45	3.18	0.47	2.18	0.39
Highly burned out	24.97	9.09	14.23	1.43	11.13	0.92	7.13	0.96

### The role of teaching behaviors in predicting profiles among children

To determine the role of student characteristics and perceived teaching behaviors in predicting the five identified profiles, a follow-up logistic regression analysis was conducted. Within the regression model, six variables regarding teaching behaviors (e.g., choices, understanding, suppression) and three factors on student characteristics (i.e., region, grade, and gender) were added in the final model as covariates. Adding these covariates in the model resulted in similar group membership to those in the final LPA model concerning the size and interpretation of the latent groups.

In general, the results with the covariates (Table 4) showed that students who believed teachers tend to provide choices, cultivate understanding and interest, and permit criticism and independent thinking from students were more likely to belong to the engaged rather than the burned out groups and to be highly engaged rather than the moderately engaged group. Additionally, those who rated teaching behaviors as suppressing (criticism & independent viewpoint) and forcing (meaningless activities) were more likely to be in the burned out groups than the engaged groups.

**Table 4:** Estimated log odds for teaching styles and student characteristics predicting latent profile membership

	C1 vs. C5	C2 vs. C5	C3 vs. C5	C4 vs. C5	C1 vs. C4	C2 vs. C4	C3 vs. C4	C1 vs. C3	C2 vs. C3	C1 vs. C2
	Β	β	β	β	β	β	β	β	β	β
Choices	− 0.18	− 0.22	− 0.05	− 0.052	− 0.12	− 0.17*	0.00	− 0.13	− 0.17*	0.04
Understand	0.04	0.21	0.26	0.328	− 0.29*	− 0.12	− 0.07	− 0.22	− 0.04	− 0.18
Criticism	0.07	− 0.07	− 0.14	0.239	− 0.17	− 0.31**	0.38***	0.22	0.08	0.14
Intrude	0.23	0.11	0.14	0.159	0.07	− 0.05	− 0.02	0.09	− 0.04	0.12
Suppress	− 0.11	− 0.20	− 0.32*	− 0.31*	0.21*	0.12	− 0.01	0.22*	0.12	0.09
Meaningless	− 0.21*	0.32**	0.60***	0.54***	0.33***	0.22**	− 0.06	0.39***	0.28**	0.11
Region = 1	− 0.59	− 0.44	0.36	0.88	1.47***	1.32***	− 0.52*	− 0.95**	0.80**	− 0.15
4th grade	− 0.71	− 0.99	− 1.13	− 1.218*	0.51	0.22	0.09	0.42	0.14	0.28
5th grade	− 0.51	− 0.30	− 0.21	0.393	− 0.90*	0.70**	− 0.60*	− 0.3	− 0.10	− 0.21
Male	− 0.43	− 0.63	− 0.48	− 0.78	0.351	0.15	0.30	0.05	− 0.15	0.20

Groups printed in italics are the reference group

*C1* Moderately burned out; *C2* Slightly burned out; *C3* Moderately engaged; *C4* Highly engaged; *C5* Highly burned out, Region 1 = urban school

*p < .05; **p < .01; **p < .001

### Associations between students’ characteristics and engagement profiles

In terms of student characteristics, those who identified themselves as attending urban schools were more likely to belong to the burned out groups rather than the engaged groups and more likely to belong to the stressed groups rather than the burned out groups than their suburban counterparts. Finally, according to the odds ratio (as indicated in Table 4), students in lower grades (i.e., 4th grade) were generally more likely to belong to the engaged groups than to the burned out groups, but higher-grade students (e.g., 5th grade) were more likely to belong to the slightly to moderately burned out groups than to the engaged groups and more likely to belong to the moderately engaged group than to the highly engaged group. Interestingly, we did not find any significant gender differences in predicting those five profile memberships. Taken together, the results show that students attending urban schools and students in higher grades have a higher likelihood of being burned out than those attending suburban schools and lower grades.

## Discussion and limitations

### The latent profiles of student engagement and school burnout

Using a person-oriented approach, this study examined the latent profiles of student engagement and study-related burnout (i.e., exhaustion, cynicism, and inadequacy), as well as the role of student characteristics and perceived teaching behaviors (i.e., providing choice, permitting criticism, and suppressing independent viewpoints) in predicting latent profile memberships. The results showed that five profiles could be identified among elementary school students: moderately burned out (12%), slightly burned out (44%), moderately engaged (15%), highly engaged (25%), and highly burned out (4%). To date, this is one of the earliest efforts made to understand the current generation of Chinese children’s engagement and burnout profiles. Nevertheless, the result is slightly shocking since it seems that two out of three students in this study belong to the profile groups of burned out. Even though a large majority of them reported mild burnout symptoms, from the perspective of developmental psychology, this is a trend that cannot be ignored; it may indicate a poor school fit and overall adjustment to the school environment. Additionally, school burnout is likely to affect adolescents’ long-term academic careers [15]. Due to their dissatisfaction, these students may not be inspired to complete their studies, putting them at risk of dropping out of school [10].

Interestingly, earlier person-centered studies also identified a similar portion of engaged and burned out students (e.g., 8, 20). For example, among secondary school students, a study with a Nordic sample reported a 5.5% low engagement/high burnout profile [19], just slightly above the level in this study. Moreover, in the context of Finnish (high school) students, the percentage of the burned out profile is 19% [8], which is similar to our rate of 17% when the moderately (12%) and highly burned out (4%) groups are combined.

### The predictive role of teacher behaviors on student engagement profiles

There is ample evidence showing that students’ perceived teacher support and care have the potential to facilitate engagement [32] and protect against school burnout ^61^. Students with the best fit with school reported receiving effective support at school [32, 60], particularly from teachers [61]. In this study, as expected, students who believed teachers were willing to provide choices, cultivate understanding and interest, and permit criticism and independent thinking from students were likelier to belong to the engaged groups. In contrast, those who rated teacher behaviors as suppressing (criticism & independent viewpoint) and forcing (meaningless activities) were more likely to belong to the burned out groups than to the engaged groups. This finding is consistent with previous studies [43, 44]. From the perspectives of early studies [43, 46], those motivational teaching strategies can be further classified into autonomy-supportive and autonomy-suppressing teacher behaviors.

Interestingly, among the autonomy-supportive teacher behaviors (i.e., providing choices, cultivating understanding and interest, and permitting criticism and independent thought), allowing criticism and supporting independent thinking has the strongest predictive effect on students’ profile membership of engagement. This can probably be explained by two reasons. First, interest is the most important motivational factor for learning. When teachers are unable to make learning tasks more interesting, criticism from students may encourage teachers to provide a more convincing rationale for learning activities, which leads to a better evaluation of tasks and student satisfaction. Second, critical/independent thinking, as a crucial 21st-century skill, is emphasized both by educators and valued by students. As a result, in earlier research, supporting critical thinking in the classroom has been found to be associated with student engagement [62, 63]. Similarly, for autonomy-suppressing, forcing meaningless and uninteresting activities seems to be the most active predictor of burnout profile membership. This is consistent with several previous studies [64], yet conflicting with others [43, 59], since they suggested that suppressing criticism was expected to be the subtype of autonomy-suppressing behavior that is the best predictor of negative affect and lack of engagement for early adolescents. This is explainable since students’ interest and engagement in school are declining, especially in STEM-related subject fields (European Commission, 2017). Research has found that presence increases the likelihood of learners engaged in their learning and problem solving [65, 66]. Additionally, the absence of interest may lead to burnout symptoms [67]. When teachers compel things that students find boring or meaningless, the students have low levels of motivation. As a result, students end up seeking more interesting tasks in the academic world and becoming burned out.

### Associations between student characteristics and the profiles

In addition to teacher behaviors, we investigated the associations between student characteristics (gender, grade and region) and profile memberships. Logistic regression indicated that grade is the most significant predictor of students’ engagement and burnout subgroups. Specifically, students from urban schools were more likely to belong to the burned out groups, unlike rural school students, who were likely to belong to burnout groups. Additionally, children of lower grades were more likely to be engaged in profile memberships than burned out children. However, differences did not exist between boys and girls.

Grade is a crucial student-level factor in engagement and burnout. Students in this study featured middle to late elementary school years (grades 4 to 6). Early research has shown that as students begin to emerge in late elementary school and continue into secondary educational experiences, their motivation and engagement with school tend to diminish [52, 54]. From the perspective of cognitive science, students’ perceptions of their motivations and capabilities (as a key antecedent of their engagement) in learning emerge around the age of 6 and continue to differentiate into early adolescence [3, 68]. Such within-individual level fluctuations could contribute to the significant relationship between grade level and the likelihood of burned out profiles. In addition, the relationship between students’ grades and the greater likelihood of a burned out profile may be explained by changes in students’ level of engagement and changes in engagement across different contexts near the end of their elementary school years. One is related to academic self-efficacy [69], and the other may be changes in their perceived school climate or social support (i.e., teacher support, peer support), teacher-student relations, and increases in the high-stakes nature of grades and test scores [70–72]. This situation is especially true for Chinese students since late elementary school years feature competition with peers to enter secondary schools [73].

For gender, even though we expected that there might be significant differences in favor of girls being more engaged than their boy counterparts across the elementary and secondary school period [32, 74, 75], no significant gender difference was found among the identified memberships. This, however, is in line with the results of [70], who also did not observe such differences. Most likely, the lack of significant gender differences can be explained by theories on gender similarities. For instance, one comprehensive review study [76] synthesized more than 5000 individual studies (involving approximately 7 million people) and found that both girls’ and boys’ psychological traits and cognitive abilities were quite similar. As more precise and effective educational interventions are implemented, it is possible to argue that gender differences, if any, are diminishing. This gender similarity issue is also an interesting opportunity for future studies.

Regarding school region, in this study, we found that students who studied in an urban area were likelier to belong to the burned out groups than to the engaged groups. Scholars [70] contend that students attending schools in urban environments may experience an intensive level of disengagement (or burnout) as a result of inequitable learning opportunities, mismatching student–teacher numbers, and chronic risk factors such as lack of resources and health disparities that have something to do with poverty [53]. In the current Chinese education system, urban schools are more likely to be heavily populated, and intensive competition exists between schools and pupils. Teachers tend to assign extracurricular tasks to already exhausted children. As a result, maladjustment to schools, such as cynicism and inadequacy, may occur. Several studies have highlighted this issue [55, 77].

### limitations and further directions

Limitations exist in almost every study. Our study is not an exception. First, there were restrictions on the scope of this study: we discovered that students who regarded their teachers’ pedagogical approaches as autonomy-supportive were more likely to be engaged rather than burned out. However, readers should keep in mind that student engagement (and burnout) are complex, fluid processes involving a dynamic system of social and psychological constructs and synergistic processes [1]. That being said, future research should investigate these topics in greater depth by incorporating more social and psychological variables, such as socioemotional skills, perseverance, and academic buoyancy. In addition, this study employed questionnaire replies from students. Estimates based on self-reports of students’ school experience may be unreliable because individuals may define content differently or incorrectly recall the frequency/levels with which they view their attitudes and teachers’ behaviors [78, 79]. Despite the fact that the majority of significant aspects of the environment can only be understood by evaluating how pupils perceive them [1], a better result can be acquired from different perspectives. One example is inviting trained observers to rate teachers’ instructional styles and students’ ‘ngagement (i.e., behavioral engagement) and combining this with their subjective feelings of engagement and burnout. Furthermore, the design of the study was cross-sectional, and it is important to be aware of the predictive limitations of cross-sectional studies. For example, there is often no evidence of a temporal relationship between exposure and outcome due to the simultaneous assessment of exposure and outcome. Thus, in future studies, it is crucial to consider the developmental aspect of school burnout and engagement profiles and how they change according to student characteristics [21, 80]. Finally, in this study, we measured burnout from exhaustion, cynicism, and inadequacy. However, they were mainly focused on the emotional facets of the syndrome. In addition, there have been recent developments that examine burnout from a multidimensional approach. Some have developed a multicomponent measure to detect student burnout by simultaneously measuring cognitive, behavioral, and emotional difficulties, which consists of four subscales: exhaustion, mental distance, cognitive impairment, and emotional impairment [81]. Such a multidimensional perspective of school burnout should be an opportunity for future study. Finally, we repeated several earlier research findings. Current studies are inconsistent at a tremendous scale. Further investigation is needed to promote the discussion on how providing children with voice and choices (or vice versa) could contribute to engagement profiles and buffer against potential maladjustment in schools, such as burnout symptoms and academic stress. This is an important step toward school dropout interventions.

## Conclusion

Using a sample from elementary-level students and a person-centered analysis approach, this study supported the idea that student characteristics (i.e., grade and school region) and the active nature of teachers’ roles have a predictive effect on elementary school children’s engagement and burnout. Specifically, we found the following: (1) students of higher/late elementary school years were linked with a high likelihood of belonging to burned out membership profiles; (2) children who identified themselves as attending urban schools were more likely to be in the burnout profiles than in the engaged profiles; and (3) when students perceived teachers as being autonomy supportive in class, they were more likely to be in the engagement profiles. Taken together, the findings underscore the importance of both teachers’ roles and the contextual factors that may contribute to students’ school well-being (e.g., engagement) and (mal)adjustment (e.g., burnout). Moreover, it further suggests that autonomy support is crucial for adolescents and children in early school years [40]. Therefore, more in-depth research work is needed on this topic, especially for burnout symptoms, given that school burnout is likely to have negative effects on teenagers’ long-term school careers [15] and because of disaffection, these students are at risk of dropping out of school and the educational system [10]. Based on such significance, future research is needed to better understand students’ learning engagement and burnout and effective interventions.

## Data Availability

The datasets used and/or analyzed during the current study are available from the corresponding author on reasonable request.
